# Quantification of Expected Information Gain in Visual Acuity and Contrast Sensitivity Tests

**DOI:** 10.21203/rs.3.rs-3031340/v1

**Published:** 2023-06-09

**Authors:** Zhong-Lin Lu, Yukai Zhao, Luis Andres Lesmes, Michael Dorr

**Affiliations:** 1 Division of Arts and Sciences, NYU Shanghai, Shanghai, China; Center for Neural Science and Department of Psychology, New York University, New York, USA; NYU-ECNU Institute of Brain and Cognitive Neuroscience, Shanghai, China.; 2 Center for Neural Science, New York University, New York, USA; 3 Adaptive Sensory Technology Inc., San Diego, CA, USA

**Keywords:** Information gain, Visual Acuity, Contrast Sensitivity Function

## Abstract

We introduce expected information gain to quantify measurements and apply it to compare visual acuity (VA) and contrast sensitivity (CS) tests. We simulated observers with parameters covered by the visual acuity and contrast sensitivity tests and observers based on distributions of normal observers tested in three luminance and four Bangerter foil conditions. We first generated the probability distributions of test scores for each individual in each population in the Snellen, ETDRS and qVA visual acuity tests and the Pelli-Robson, CSV-1000 and qCSF contrast sensitivity tests and constructed the probability distributions of all possible test scores of the entire population. We then computed expected information gain by subtracting expected residual entropy from the total entropy of the population. For acuity tests, ETDRS generated more expected information gain than Snellen; scored with VA threshold only or with both VA threshold and VA range, qVA with 15 rows (or 45 optotypes) generated more expected information gain than ETDRS. For contrast sensitivity tests, CSV-1000 generated more expected information gain than Pelli-Robson; scored with AULCSF or with CS at six spatial frequencies, qCSF with 25 trials generated more expected information gain than CSV-1000. The active learning based qVA and qCSF tests can generate more expected information than the traditional paper chart tests. Although we only applied it to compare visual acuity and contrast sensitivity tests, information gain is a general concept that can be used to compare measurements and data analytics in any domain.

## INTRODUCTION

Measurement is the foundation of the scientific method.^1–4^ From physics to biology, to engineering, to social sciences and medicine, data obtained from measurement is used to test hypothesis, construct theory, evaluate performance, and generate diagnosis.^5–9^ In this study, we introduce a method to compare measurements in terms of expected information gain and apply it to compare visual acuity (VA) and contrast sensitivity (CS) tests.

Quality metrics have been developed to evaluate individual measurements and the agreement between two unidimensional measurements.^10–12^ However, it remains a challenge to compare novel measurements with different dimensionalities to current gold standards. The potential advantages of a new measurement are difficult to evaluate if measurements from the same patients differ because the measurement yields outcomes in more dimensions. Visual acuity is the most prominent function vision measure that is widely used in diagnosing and managing visual disease, evaluating treatment efficacy, and setting professional standards.^13–15^ The gold standard ETDRS chart^16^ consists of rows of five equal-sized optotypes in descending order and generates a unidimensional VA threshold score for each patient. The newly developed qVA^17, 18^, however, tests patients with three equal-sized optotypes in each trial and generates a two-dimensional score, VA threshold and VA range, for each patient. The VA threshold scores of the same patient from the two tests are not exactly the same because of the different optotype arrangements, and comparing only VA threshold scores from the two tests would have ignored the additional VA range score obtained in qVA. The contrast sensitivity function (CSF), a more comprehensive characterization of spatial vision, is increasingly used in clinical research and clinical trials.^19, 20^ Like visual acuity, various instruments have also been developed to measure contrast sensitivity,^21–23^ using different optotypes and generating scores with different dimensionalities. The Pelli-Robson test^22^ uses unfiltered Sloan letters and generates a unidimensional contrast sensitivity score at one spatial frequency, the CSV-1000 test^24^ uses windowed sinusoidal gratings and generates a four-dimensional contrast sensitivity score at four spatial frequencies, and the qCSF^23, 25^ uses filtered Sloan letters and generates both a unidimensional area under the log CSF (AULCSF) score and a six-dimensional contrast sensitivity score at six spatial frequencies. How to compare CS instruments with different optotypes and outcome dimensionalities is very challenging.

We propose applying expected information gain^26^ (also known as expected mutual information) to quantify measurements. From an information theory perspective,^27–29^ measurement is “A set of observations that reduce uncertainty where the result is expressed as a quantity.”^30^ The amount of expected information gain from a measurement can be obtained by comparing the uncertainties before and after the measurement.

**Figure 1.**
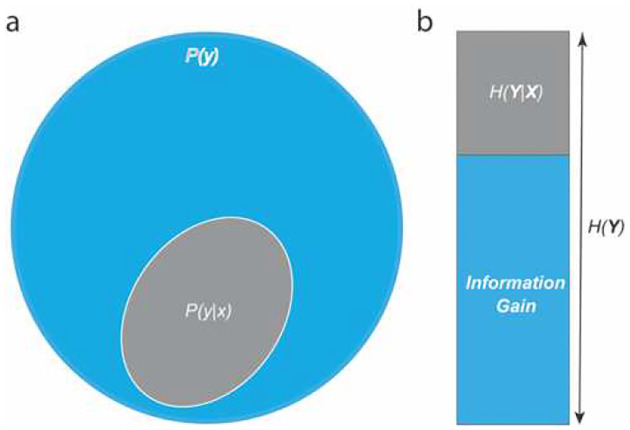
(a) Distribution of *Y*, representing the distribution of all potential measurement outcomes, and outcome distribution *P*(*y*|*x*) from measurement on a single *x*. (b) Expected information gain is the difference between *H*(*Y*) and the expected residual entropy *H*(*Y*|*X*).

**Figure 2.**
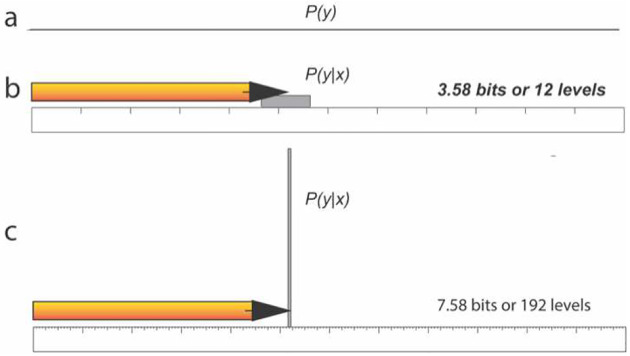
An illustrations of information gain with two footlong rulers, one with a one inch unit and the other with a 1/16 inch unit. (a) A uniformly distributed *P*(*y*). (b) *P*(*y*|*x*) and expected information gain of the ruler with a 1-inch unit. (c) *P*(*y*|*x*) and expected information gain of the ruler with a 1/16-inch unit.

We begin with the measurement population, characterized by a random variable *X* with probability density function *P*(*x*). Suppose for each sample *x* from *X*, the measurement outcome variable *Y* has a probability density distribution *P*(*y*|*x*) (Figure 1a). The probability density function of *Y* (Figure 1a), *P*(*y*) = ∫ *P*(*y*|*x*) *P*(*x*)*dx*, characterizes the probability of all potential outcomes from the measurement. We then use Shannon entropy of *Y*, *H*(*Y*) = − ∫ *P*(*y*)*log*_2_(*P*(*y*))*dy*, to quantify the amount of information in the outcome variable *Y*, and the expected entropy of *P*(*y*|*x*), *H*(*Y*|*X*) = −∫ *P*(*x*)*dx* ∫ *P*(*y*|*x*)*log*_2_(*P*(*y*|*x*))*dy*, to quantify the expected residual uncertainty after performing measurement on *X*. Finally, we define the difference between *H*(*Y*) and *H*(*Y*|*X*) as the expected information gain from the measurement: *IG*(*Y*|*X*) = *H*(*Y*) − *H*(*Y*|*X*) (Figure 2b).

Information gain has been widely used in machine learning as a basic criterion to optimize the decision tree, where the feature with the highest value of information gain is used to split a node of the tree.^31^ It’s also been used in active learning algorithms to select the optimal stimuli.^23, 32, 33^ However, the focus has been on relative information gain among different features or stimuli during learning, but not on the amount of expected information gain from measurements of a population.

We illustrate the concept of expected information gain using measurement with two rulers (Figures 2bc). For a ruler with a total length *L* and unit Δ, the posterior distribution from the measurement of an object with length *x* is: *P*(*y*│*x*) = *U*(*x* − Δ/2, *x* + Δ/2), where *U*(*a*, *b*) is a uniform distribution with boundaries *a* and b. If the ruler is used to measure objects with length between 0 and *L* with equal probability, *P*(*x*) = *U*(0, *L*), then:

(1a) $$P(y)=\int_{0}^{L}\text{U}\left(\text{x}-\frac{\Delta }{2},\text{x}+\frac{\Delta }{2}\right)U(0,L)dx=U(0,L),$$

(1b) $$H(Y)=-\int_{0}^{L}U(0,L)log_{2}(U(0,L))dy=log_{2}(L),$$

(1c) $$H(Y|X)=-\int_{0}^{L}U(0,L)dx\int_{0}^{L}U\left(x-\frac{\Delta }{2},x+\frac{\Delta }{2}\right)log_{2}\left(U\left(x-\frac{\Delta }{2},x+\frac{\Delta }{2}\right)\right)dy=log_{2}(\Delta ),$$

(1d) $$IG(Y|X)=log_{2}(L/\Delta ).$$

For two footlong rulers, one with a one inch unit and the other with a 1/16 inch unit, the expected information gains are ${\text{log}}_{2}\left(\frac{12}{1}\right)=3.58\text{ bits}$ and ${\text{ log}}_{2}\left(\frac{12}{1/16}\right)=7.58\text{ bits}$, corresponding to 12 and 192 length classes, respectively, consistent with the number of units on the rulers.

The outcome distributions of a ruler are uniform. We can also derive expected information gain of a unidimensional measurement with a normal outcome distribution: $P(y|x)=\frac{1}{\sqrt{2\pi }\sigma }e^{-\frac{{\left(y-\mu (x)\right)}^{2}}{2\sigma^{2}}}$, where *μ*(*x*) is the expected value of x. If both *x* and *y* are uniformly distributed in an interval of length *L*, then $H(Y)=log_{2}(L),H(Y|X)=log_{2}(\sigma \sqrt{2\pi e})$, and *IG*(*Y*|*X*) = *log*_2_(*L*/(4.13*σ*)), which leads to L/(4.13*σ*) classes. Intuitively, the size of each class is 4.13*σ*, corresponding to the 98% confidence interval of the outcome distribution.

Although we illustrate it in two unidimensional examples, expected information gain can be computed from measurements in any dimensionality and can be used to compare measurements with different dimensionalities. In this study, we computed expected information gain in visual acuity and contrast sensitivity tests. Visual acuity is the most prominent function vision measure that is widely used in diagnosing and managing visual disease, evaluating treatment efficacy, and setting professional standards.^13–15^ The contrast sensitivity function (CSF), a more comprehensive characterization of spatial vision, is increasingly used in clinical research and clinical trials.^19, 20^ Various instruments have been developed to measure visual acuity^16, 17, 34, 35^ and contrast sensitivity^21–23^. Many studies have been conducted to evaluate their accuracy, test-retest variability, sensitivity, and specificity.^18, 36–48^ However, because different tests use different optotypes or optotype arrangements and generate scores with different dimensionalities, it has been difficult to compare them.

Specifically, we computed expected information gain from the Snellen chart,^34^ ETDRS chart,^16^ and qVA test^17^ for visual acuity assessment, and Pelli-Robson chart,^22^ CSV-1000 chart (Vector Vision, Houston, Texas),^24^ and qCSF test^23, 25^ for contrast sensitivity assessment through computer simulations. We computed and compared the amount of expected information gain and number of classes from these measurements in a population with a uniform distribution that covers the entire range of all possible observers specified in the tests and a population with a distribution derived from observed experimental data.

## METHODS

### Apparatus

All the simulations and analyses were conducted on a Dell computer with Intel Xeon W-2145 @ 3.70 GHz CPU (8 cores and 16 threads) and 64 GB installed memory (RAM) with Matlab R2019a (MathWorks Corp., Natick, MA, USA) and R (R Core Team, 2020).

### Visual Acuity Tests

#### Simulated observers.

**Figure 3.**
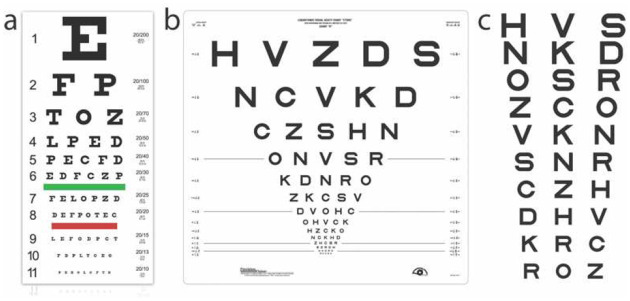
(a) A Snellen chart (Image courtesy of Precision Vision, Inc.) (b) An ETDRS chart (Image courtesy of Precision Vision, Inc.) (c) A subset of potential stimuli in qVA.

We conducted two simulations using the Snellen, ETDRS, and qVA tests (Figure 3). In Simulation 1, we simulated 1386 observers from a uniform distribution of VA threshold $\left(\theta_{\text{Threshold}}^{VA}\right)$ and VA range $\left(\theta_{\text{Range}}^{VA}\right)$, with $\theta_{\text{Threshold}}^{VA}\in [-0.3,1.0]$ logMAR and sampled every 0.02 logMAR, and $log_{10}\left(\theta_{\text{Range }}^{VA}\right)\in [-1.0,0]$ and sampled every 0.05 *log*_10_ units. In Simulation 2, we simulated 1386 observers from the population distribution of VA threshold and VA range derived from an existing qVA dataset of 14 eyes tested with Bangerter foils.^18, 49^.

#### Visual Acuity Behavioral Function.

For each simulated observer, the discriminability (*d*′) for an optotype of size *s* is described by the visual acuity behavioral function (Patent No. US 10758120B2):^17^

(2) $$d^{\prime }\left(s|\theta^{VA}\right)={\text{log}}_{10}(6)+\frac{\omega }{2\theta_{\text{Range }}^{VA}}\left(s-\theta_{\text{Threshold }}^{VA}\right)-\frac{1}{2}{\text{log}}_{10}\left(8+10^{\frac{\omega }{\theta_{\text{Range }}^{VA}}\left(s-\theta_{\text{Threshold }}^{VA}\right)}\right),$$

where $\theta^{VA}=\left(\theta_{\text{Threshold}}^{VA},\theta_{\text{Range}}^{VA}\right)$, $\theta_{\text{Threshold}}^{VA}$ is VA threshold, corresponding to the optotype size at *d*′=2, $\theta_{\text{Range}}^{VA}$ is the VA range of the behavioral function, that is, the range of optotype sizes that covers *d*′=1 to *d*′ = 4 performance levels, and *ω* = log_10_ 35 − log_10_ 1.25.

In these visual acuity tests, observers identify optotypes from the 10 Sloan letters. We can compute the probability of obtaining *m* correct responses in the 10-alternative forced identification task in a row of *M* randomly sampled optotypes from their *d*’ function (Patent No. US 10758120B2):^17, 18^

(3) $$\text{p}\left(m|s,M,\theta^{VA}\right)=f\left(m,M,d^{\prime }\left(s|\theta^{VA}\right)\right)\text{, }$$

where *f*(.) is derived from signal detection theory, taking into account of each chart design, i.e., the number of optotypes in a row and whether optotypes in each row are sampled from the 10 Sloan letters with or without replacement.

#### Snellen Chart.

The Snellen chart (Figure 3a) has 11-rows, with 1, 2, 3, 4, 5, 6, 7, 8, 8, 8, and 9 optotypes in each row and optotype size descending from 1.0 to −0.3 logMAR. Each simulated observer was tested with the standard procedure.^50^ The probability of correctly identifying *m* optotypes in a row is determined by Eq. 3 with varying *M* across rows. Starting from the top row, the observer must correctly identify at least half of the optotypes on a row before proceeding to the next row. If they can’t identify the optotype on the top row, the acuity score was 1.1 logMAR; otherwise, the acuity score is equal to the size of the optotypes in the last row with at least 50% correct identification. The acuity score could therefore take 12 potential values. For each simulated observer *x*_*i*_, we repeated the test 1000 times to obtain the distribution of test scores *P*(*y*_*j*_|*x*_*i*_), where *j* = 1, …, 12.

#### ETDRS Chart.

The ETDRS chart (Figure 3b) has 14 five-optotype rows, with optotype size descending from 1.0 to −0.3 logMAR. Each simulated observer was tested with the standard procedure.^16, 50^ The probability of correctly identifying *m* optotypes in a row is determined by Eq. 3 with *M* = 5. Four different termination rules were simulated. Starting from the top row, the test could stop after the observer makes three, four, or five mistakes in identifying the optotypes in a row or continue until the observer is tested with the entire chart. The acuity score is computed as 1.1 − 0.02*n*, where *n* is the number of correctly identified optotypes, with 71 potential values. For each simulated observer *x*_*i*_, we repeated the test 1000 times to obtain the distribution of test scores *P*(*y*_*j*_|*x*_*i*_), where *j* = 1, …, 71.

#### qVA Test.

The qVA (Figure 3c) is a Bayesian active learning visual acuity test.^17^ Its stimulus space consists of optotypes of 91 linearly spaced sizes from −0.5 to 1.3 logMAR, with a 0.02 logMAR resolution. Starting with a weak prior distribution of VA threshold and VA range in a two-dimensional space that has 700 linearly spaced VA thresholds (between −0.5 and 1.3 logMAR) and 699 log-linearly spaced VA ranges (between 0.1 and 1.5 logMAR), it uses an active learning procedure to test the observer with the optimal stimulus in each trial and generates the posterior distribution of VA threshold and range. Each observer was tested with 5, 15, or 30 rows (corresponding to 15, 45, or 90 optotypes). The probability of correctly identifying *m* optotypes in a row is determined by Eq. 3 with *M* = 3. We computed the mean VA threshold and range from their posterior distributions in each test and quantized them into 86 and 56 discrete scores with a 0.02 logMAR resolution, with a total of 4816 potential combinations. For each simulated observer *x*_*i*_, we repeated the test 1000 times to obtain the two-dimensional distribution of test scores *P*(*y*_*j*_|*x*_*i*_), with *j* = 1, …,4816. We also computed the distribution of VA threshold *P*(*y*_*Threshold*,*j*_|*x*_*i*_) by marginalizing P(*y*_*j*_|*x*_*i*_):

(4) $$P\left(y_{\text{Threshold, }j}|x_{i}\right)=\sum_{\text{range}}P\left(y_{j}|x_{i}\right),$$

where j=1,…,86.

#### Information Gain.

We first computed P(*y*_*j*_) from *P*(*y*_*i*_|*x*_*i*_) for each test:

(5) $$P\left(y_{j}\right)=\frac{1}{I}\sum_{i=1}^{I}P\left(y_{j}|x_{i}\right),$$

where I=1386 in both simulations. We then computed the total entropy of each test of a population:

(6) $$H(Y)=-\sum_{j=1}^{J}P\left(y_{j}\right)log_{2}\left(P\left(y_{j}\right)\right),$$

where J=12, 71, 86, 4816 for the Snellen, ETDRS, VA threshold from qVA, and VA threshold and VA range from qVA, the expected residual entropy:

(7) $$H(Y|X)=-\frac{1}{I}\sum_{i=1}^{I}\sum_{j=1}^{J}P\left(y_{j}|x_{i}\right)log_{2}\left(P\left(y_{j}|x_{i}\right)\right)\text{, }$$

and, finally expected information gain:

(8) IG(Y∣X)=H(Y)−H(Y∣X).

### Contrast Sensitivity Tests

#### Simulated observers.

We conducted two simulations using the Pelli Robson chart, CSV-1000 chart, and qCSF test (Figure 4). In Simulation 1, we simulated 1911 observers from a uniform distribution of peak gain $\left(\theta_{PG}^{CSF}\right)$, peak spatial frequency $\left(\theta_{PF}^{CSF}\right)$, and band width $\left(\theta_{BH}^{CSF}\right)$, with $log_{10}\left(\theta_{PG}^{CSF}\right)\in [0.3,2.3]$ sampled every 0.10 log_10_ units, $log_{10}\left(\theta_{PF}^{CSF}\right)\in [-0.3,0.9]$ sampled every 0.10 log_10_ units, and $log_{10}\left(\theta_{PF}^{CSF}\right)\in [-0.3,0.9]$ sampled every 0.05 log_10_ units. In Simulation 2, we simulated 1911 observers from the population distribution of CSF parameters derived from two existing qCSF datasets, one consisted of 112 eyes tested in three luminance conditions^39^ and the other of 14 eyes tested with Bangerter foils^18^, using a hierarchical Bayesian model.^49, 51^

#### Letter and Grating Contrast Sensitivity Functions.

The letter contrast sensitivity function, which specifies contrast sensitivity *S*_*lette*_(*f*) for filtered letters of different sizes at center spatial frequency *f*, can be described with a log parabola function with three parameters $\theta^{CSF}=\left(\theta_{PG}^{CSF},\theta_{PF}^{CSF},\theta_{BH}^{CSF}\right)$:^23, 52, 53^

(9) $${\text{log}}_{10}\left(S_{\text{letter}}\left(f|\theta^{CSF}\right)\right)={\text{log}}_{10}\left(\theta_{PG}^{CSF}\right)-\frac{4}{{\text{log}}_{10}(2)}{\left(\frac{{\text{log}}_{10}(f)-{\text{log}}_{10}\left(\theta_{PF}^{CSF}\right)}{\theta_{BH}^{CSF}}\right)}^{2},$$

where $\theta_{PG}^{CSF}$ is the peak gain, $\theta_{PF}^{CSF}$ is the peak spatial frequency (cycles/degree), and $\theta_{BH}^{CSF}$ is the bandwidth (octaves) at half-height. For an observer with peak gain $\theta_{PG}^{CSF}$, peak spatial frequency $\theta_{PF}^{CSF}$, and bandwidth $\theta_{PF}^{CSF}$, the probability of correct identification of a bandpass-filtered optotype with contrast *c* and center spatial frequency *f* is described with a Weibull psychometric function:^25^

(10) $$p\left(\text{correct}|\theta^{CSF},f,c\right)=g+\left(1-g-\frac{\lambda }{2}\right)\left[1-\text{exp}\left[-{\left(c\times S_{\text{letter}}\left(f|\theta^{CSF}\right)\right)}^{b}\right]\right]\text{, }$$

where *g* is the guessing rate, *λ*=0.04 is the lapse rate, and *b* determines the steepness of the psychometric function. Because they both use a 10-alternative forced identification task, *g* = 0.1 and *b* = 4.05 for the Pelli-Robson chart and qCSF test.

The grating contrast sensitivity function, which specifies contrast sensitivity *S*_*grating*_(*f*) for gratings of the same size at spatial frequency *f*, needs to be corrected for the increased number of cycles with increasing spatial frequency.^54^ For the grating stimuli used in the CSV-1000:

(11) $${\text{log}}_{10}\left(S_{\text{grating}}\left(f|\theta^{CSF}\right)\right)={\text{log}}_{10}\left(\theta_{PG}^{CSF}\right)-\frac{4}{{\text{log}}_{10}(2)}{\left(\frac{{\text{log}}_{10}(f)-{\text{log}}_{10}\left(\theta_{PF}^{CSF}\right)}{\theta_{BH}^{CSF}}\right)}^{2}+{\text{log}}_{10}(0.539f).$$

For the yes/no task in the first column of the CSV-1000 test, we used a high-threshold model. That is, the simulated observer says yes if the stimulus contrast > threshold (=1/*S*_*grating*_(*f*|*θ*^*CSF*^)). The simulated observer says no otherwise. For the two-alternative forced choice task in CSV-1000, we replace *S*_*letter*_(*f*|*θ*^*CSF*^) with *S*_*grating*_(*f*|*θ*^*CSF*^) and set *g* = 0.5, *b* = 3.06 in Equation 9 to compute the probability of making a correct response.

#### Pelli-Robson Chart.

The Pelli-Robson chart (Figure 4a) consists of 16 optotype triplets of the same size and log-linearly spaced contrast between 0.56% and 100%.^24^ At a viewing distance of 3 m, the center frequency of the optotypes is 3 c/d. The probability of correctly identifying each optotype in the chart is determined by Eq. 10. Starting from the top row, the test proceeds to the next triplets only if the observer correctly identifies at least two of the three optotypes in the current triplet. The contrast sensitivity of the observer is determined by the lowest contrast *c*_*lowest*_ at which they correctly identify at least two of the three letters in the triplet: *S*_*letter*_(3*c*/*d*) = −*log*_10_(*c*_*lowest*_), with 17 potential contrast sensitivity scores. For each simulated observer *x*_*i*_, we repeated the test 1000 times to obtain the distribution of test scores *P*(*y*_*j*_|*x*_*i*_), where *j* = 1, …, 17.

#### CSV-1000 Chart.

The CSV-1000 chart (Figure 4b) consists of contrast sensitivity tests at four spatial frequencies: 3, 6, 12, and 18 cycles/degree. Each test consists of 17 stimuli arranged in nine columns, with a single high-contrast vertical sinewave grating in the first column, and two test patches in the remaining eight columns, of which only one contains a vertical sinewave grating. The gratings are arranged with decreasing contrast from left to right, with contrast from −0.70 to −2.08, −0.91 to −2.29, −0.61 to −1.99, and −0.17 to −1.55 log10 units in the four rows. Going through all four rows starting from the top, the observer is first required to perform a yes/no task on the first column in each row. If the observer can’t see the stimulus in the first column, the test stops for that row and the observer’s contrast sensitivity is:

(12) $$S(f)=\{\begin{array}{c} -log_{10}\left(c_{\text{first column}}(f)\right)-0.3,f=3,6,12\\ 0.01,f=18 \end{array}.$$

If the observer can see the stimulus in the first column, they proceed to identify the location of the patch that contained the grating in each column with a three-alternative forced choice response: top, bottom, or blank. We treat blank as an incorrect response. The lowest contrast at which the observer correctly identifies the location of the grating is used to determine contrast sensitivity in the test: *S*(*f*) = −*log*_10_(*c*_*lowest*_(*f*)). The result is a four-dimensional contrast sensitivity score sampled at four spatial frequencies. Because there are 10 potential contrast sensitivity scores in each spatial frequency, there are therefore a total of 10^4^ potential contrast sensitivity functions. For each simulated observer *x*_*i*_, we repeated the test 1000 times to obtain the distribution of test scores *P*(*y*_*j*_|*x*_*i*_), where *j* = 1, …, 10^4^.

#### qCSF Test.

The qCSF (Figure 4c) is a Bayesian active learning contrast sensitivity test.^23, 25^ Its stimulus space consists of 128 log-linearly spaced contrasts (from 0.002 to 1.0) and 19 log-linearly spaced spatial frequencies (from 1.19 to 30.95 c/d). Although a four-parameter truncated log parabola has been used in the qCSF test,^23^ we removed the truncation parameter in the simulations because we didn’t score the simulated observers in very low spatial frequencies. Starting with a weak prior distribution of peak gain, peak frequency and bandwidth in a three-dimensional space that has 60 log-linearly spaced peak gains (from 1.05 to 1050), 40 log-linearly spaced peak frequencies (from 0.1 to 20 c/d), and 27 log-linearly spaced bandwidth (from 1 to 9 octaves), it uses an active learning procedure to test the observer with the optimal stimuli in each trial and computes the posterior distribution of the three CSF parameters in the afore-mentioned three-dimensional space. In each trial, three filtered optotypes with the same center spatial frequency but four, two, and one times the optimal contrast (capped at 0.9) are presented. The observer could be tested with 15, 25 and 50 trials. For each simulated observer *x*_*i*_, we repeated the test 1000 times to obtain distributions of the unidimensional AULCSF *P*(*y*_*AULCSF*,*j*_|*x*_*i*_) and the six-dimensional CSF score *P*(*y*_*CSF*,*j*_|*x*_*i*_) at six spatial frequencies (1, 1.5, 3, 6, 12 and 18 c/d). Sampling the scores at 0.05 log10 resolution, j=1, …, 57 for *y*_*AULCSF,j*_, and j=1,…, 253492 for *y*_*CSF*,*j*_.

#### Information Gain.

Equations 5–8 were used to compute P(*y*_*j*_), *H*(*Y*), *H*(*Y*|*X*), and *IG*(*Y*|*X*), with I=1911 for the two simulations, and J=17, 10000, 57, 253492 for the Pelli-Robson, CSV-1000, AULCSF from qCSF, and CSF from qCSF.

## RESULTS

### Visual Acuity Tests

The VA threshold and VA range distributions of the observers in the two simulations are shown in Figures 4ab. Figure 4c shows distributions of the test scores *P*(*y*_*j*_|*x*_*i*_) of one representative simulated observer *x*_*i*_ in the Snellen, ETDRS (3-mistake rule), and qVA test, with results from the qVA test scored as VA-only, and as both VA threshold and VA range. Figures 4de show the distributions of the test scores *P*(*y*_*j*_) of the populations in Simulations 1 and 2, respectively. Because a uniform *X* distribution is used in Simulation 1, the corresponding *P*(*y*_*j*_)′*s* from the tests are nearly uniform. On the other hand, *P*(*y*_*j*_)′*s* in Simulation 2 are more concentrated because the population is more concentrated.

The total entropy *H*(*Y*), the expected residual entropy *H*(*Y*|*X*), the expected information gain *IG*(*Y*|*X*), and the expected number of classes *N*(*Y*|*X*) from the three tests in the two simulations are listed in Table 1. The expected information gain *IG*(*Y*|*X*) is also plotted in Figure 4f. As expected, *H*(*Y*) in Simulation 2 is less than the corresponding *H*(*Y*) in Simulation 1 for all the tests because of the more concentrated *P*(*y*_*j*_) resulted from a narrower range of simulated observers. As a result, *IG*(*Y*|*X*) and *N*(*Y*|*X*) in Simulation 2 are less than those in Simulation 1.

To check the validity of the simulations, we also estimated expected information *IG*(*Y*|*X*) of the Snellen and ETDRS tests from their reported test-retest variabilities (TRV=1.96 *σ*), with the assumption that the outcome distributions are normal and have the same TRV for observers with different acuities. For both tests, the outcome scores cover −0.3 to 1.0 logMAR, expected information gain can be computed with $IG(Y|X)={\text{log}}_{2}\left(\frac{\text{L}}{4.13\sigma }\right)$, where *L* = 1.3 logMAR. For the Snellen chart, the typical TRV is 0.23 logMAR,^36^ with $\sigma =\frac{TRV}{1.96}=0.117$ logMAR, and the expected information gain is 1.4 bits. For the ETDRS chart, the typical TRV is 0.11 logMAR,^36^ with $\sigma =\frac{TRV}{1.96}=0.056$ logMAR, and the expected information gain is 2.5 bits. These estimated values are largely consistent with our results in Simulation 1.

For the qVA test, we also computed expected information gain from an existing dataset with 14 eyes tested in four Bangerter foil conditions.^18^ In this dataset, VA threshold is between −0.15 and 0.68 logMAR, and VA range is between 0.12 and 0.63 logMAR. We computed the total entropy, residual entropy and information gain based on the posterior distributions from single tests rather than distributions of test scores from repeated tests. In the qVA test, the posterior distributions from single tests are broader than those derived from repeated tests until about 45 optotypes are tested and converge to those from repeated tests afterwards.^18^ The expected information gain based on VA threshold only is 1.6, 2.6 and 2.9 bits after qVA test with 15, 45 and 90 optotypes, respectively, and the expected information gain based on VA threshold and range is 2.1, 3.2, and 3.6 bits after qVA test with 15, 45 and 90 optotypes, respectively. These results are largely consistent with those from Simulation 2.

In both simulations, ETDRS generated more expected information gain than Snellen. Scored with VA threshold only or with both VA threshold and VA range, qVA with 15 rows (or 45 optotypes) generated more expected information gain than ETDRS.

### Contrast Sensitivity Tests

**Figure 5.**
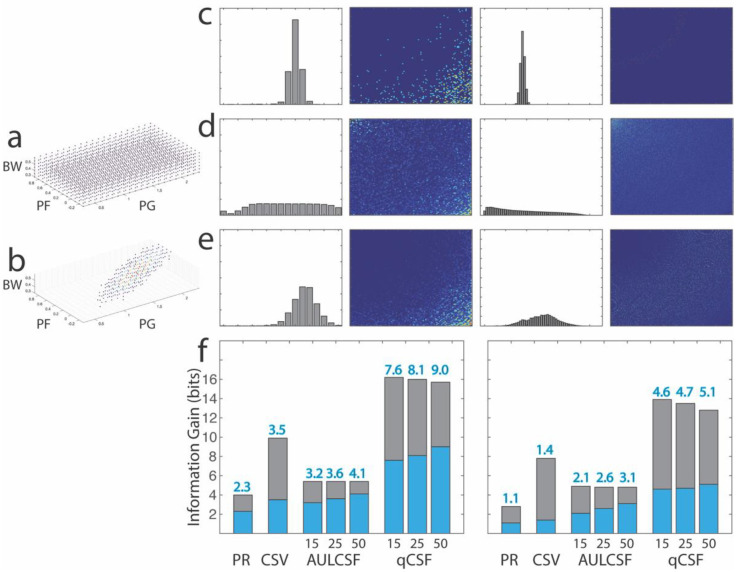
Distributions and information gain in contrast sensitivity tests. (a,b) Distributions of peak gain, peak spatial frequency, and bandwidth of the observers in Simulations 1 and 2. (c) From left to right: Distributions of the test scores *P*(*y*_*j*_|*x*_*i*_) of one representative simulated observer *x*_*i*_ in the Pelli-Robson, CSV-1000, and qCSF test (25 trials), with results from the qCSF test scored as AULCSF, and CS in six spatial frequencies. (d, e) Distributions of the test scores (*y*_*j*_) of the populations in Simulations 1 and 2, respectively. (f) Expected information gain *IG*(*Y*|*X*) of the various tests in Simulation 1 (left) and Simulation 2 (right). For qCSF, results from testing with 15, 25, and 50 trials are shown.

The peak gain, peak spatial frequency, and bandwidth distributions of the observers in the two simulations are shown in Figures 5ab. Figure 5c shows distributions of the test scores *P*(*y*_*j*_|*x*_*i*_) of one representative simulated observer *x*_*i*_ in the Pelli-Robson, CSV-1000, and qCSF test, with results from the qCSF test scored as AULCSF, and CS at six spatial frequencies. Figures 5de show the distributions of the test scores *P*(*y*_*j*_) of the populations *X* in Simulations 1 and 2, respectively. Because a uniform *X* distribution is used in Simulation 1, the corresponding (*y*_*j*_)′*s* from the tests are more uniform. On the other hand, *P*(*y*_*j*_)′*s* in Simulation 2 are more concentrated because the observers are sampled in a narrower range.

The total entropy *H*(*Y*), the expected residual entropy *H*(*Y*|*X*), the expected information gain *IG*(*Y*|*X*), and the expected number of classes *N*(*Y*|*X*) from the three tests in the two simulations are listed in Table 2. The expected information gain *IG*(*Y*|*X*) is also plotted in Figure 5f. As expected, *H*(*Y*) in Simulation 2 is less than the corresponding *H*(*Y*) in Simulation 1 in all the tests because of the more concentrated *P*(*y*_*j*_) resulted from a narrower range of simulated observers. As a result, *IG*(*Y*|*X*) and *N*(*Y*|*X*) in Simulation 2 are less than those in Simulation 1.

To check the validity of the simulations, we also estimated information *IG*(*Y*|*X*) of the Pelli-Robson test from its reported test-retest variabilities (TRV=1.96 *σ*), with the assumption that the outcome distributions are normal and have the same TRV for all the observers. For the test, the outcome scores cover 0 to 2.25 log_10_ contrast sensitivity, with a typical TRV between 0.15 and 0.20 log10 in the normal population.^55^ Therefore, *L* = 2.25 log_10_ CS, $\sigma =\frac{TRV}{1.96}$ is between 0.077 and 0.10 log_10_ CS, and the expected information gain is between 2.4 and 2.8 bits. These estimated values are largely consistent with the results in Simulation 1.

For the qCSF test, we also computed information gain from two existing datasets, one with 112 subjects tested binocularly in three luminance conditions^39^ and the other with 14 eyes tested monocularly in four Bangerter foil conditions.^18^ In this dataset, peak gain is between 0.92 and 2.27 log_10_ CS, peak spatial frequency is between 0.21 and 3.9 c/d, and the bandwidth is between 1.8 and 5.7 octaves. We computed the total entropy, expected residual entropy and expected information gain based on the posterior distributions from single tests rather than distributions of test scores from repeated tests. In the qCSF test, the posterior distributions from single tests are broader than those derived from repeated tests until about 25 trials are tested and converge to those from repeated tests afterwards.^25^ The expected information gain based on AULCSF is 2.0, 2.4, and 2.9 bits after qCSF test with 15, 25 and 50 trials, respectively, and the expected information gain based on CS at six spatial frequencies is 4.1, 4.6, and 5.3 bits after qCSF test with 15, 25 and 50 trials, respectively. Again, the results from the dataset are largely consistent with those from Simulations 2.

In both simulations, CSV-1000 generated more expected information gain than Pelli-Robson test. Scored with AULCSF or with CS at six spatial frequencies, qCSF with 25 trials generated more expected information gain than CSV-1000.

## DISCUSSION

In this study, we introduced a concept from information theory, expected information gain (or mutual information), to quantify the amount of expected information that can be obtained from measurement of a population based on the expected reduction of entropy following the measurement. It allows us to compare measurements with different dimensionalities and evaluating potential advantages of new measurements that generate higher dimensional data than the current gold standard. As the first application, we computed and compared expected information gain from the Snellen chart,^34^ ETDRS chart,^16^ and qVA^17^ visual acuity tests, and Pelli-Robson,^22^ CSV-1000,^24^ and qCSF^23, 25^ contrast sensitivity tests in two populations, one with a uniform distribution that covers the entire range of all possible observers specified in the tests and the other with a distribution derived from observed experimental data. In addition, we also compared our simulation results with estimates based on reported empirical test-retest variabilities and posterior distributions of qVA and qCSF tests in experiments. We found that, for acuity tests, ETDRS generated more expected information gain than Snellen; scored with VA threshold only or with both VA threshold and VA range, qVA with 15 rows (or 45 optotypes) generated more expected information gain than ETDRS. For contrast sensitivity tests, CSV-1000 generated more expected information gain than Pelli-Robson; scored with AULCSF or with CS at six spatial frequencies, qCSF with 25 trials generated more expected information gain than CSV-1000.

ETDRS is the current gold standard in acuity test. The qVA was developed to improve acuity testing. Although we have shown reduced VA threshold variability and excellent agreement VA threshold scores from E-ETDRS and qVA,^18, 49^ it has been impossible to provide an overall evaluation of the improvement of the new method against the gold standard because the two tests use different optotype arrangements and qVA generates two-dimensional scores while ETDRS generates unidimensional scores. Because quantification of total entropy and expected residual entropy and therefore expected information gain is independent of the optotype arrangement and the dimensionality of the outcome scores, we were able to compare the two tests and show that qVA can indeed generate additional expected information gain than ETDRS.

In contrast sensitivity tests, Pelli-Robson, CSV-1000, and qCSF use different optotypes and generate outcome scores with different dimensionality. Again, because expected information gain is independent of the optotype and the dimensionality of the outcome scores, we were able to compare the three tests and show that qCSF can indeed generate additional expected information gain than Pelli-Robson and CSV-1000.

The expected information gain is defined over a population. As a result, the value of expected information could be different for different populations. This point is demonstrated in our simulations. This dependency is very important because each measurement is associated with a target population. It’s possible that different target population may require different measurements, and different measurements may have advantages/disadvantages in different populations. When we compare them, we must consider the target population along with the measurement instruments. In this study, the same target populations were used to compare different visual acuity and contrast sensitivity tests. Consistent rank orders of the tests were obtained in two different populations.

Although we only applied it to compare visual acuity and contrast sensitivity tests, expected information gain is a general concept that can be used to compare measurements and data analytics in any domain. For example, one can compare a newly developed OCT test with existing OCT test by computing how much new expected information gain can be obtained with the new test. One can also compare structural (e.g., OCT) and functional (e.g., CSF) vision tests in terms of the amount of expected information gain they can yield in classifying patients into different target categories. One can also compare different data analytic techniques in terms of their ability to reduce uncertainty and generate expected information gain.^49, 51^

## Figures and Tables

**Figure 4.**
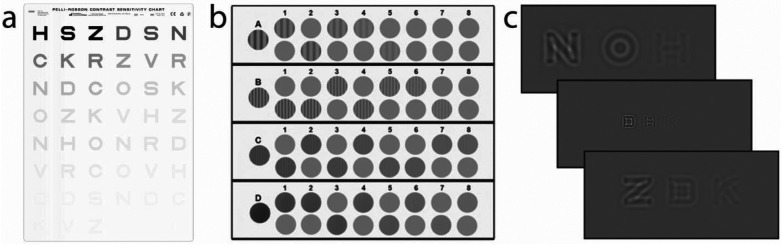
(a) A Pelli-Robson chart (Image courtesy of Precision Vision, Inc.) (b) A CSV-1000 chart (Reproduced with permission from GUARDiON Health Sciences, Inc.) (c) A subset of potential stimuli in qCSF.

**Figure 4.**
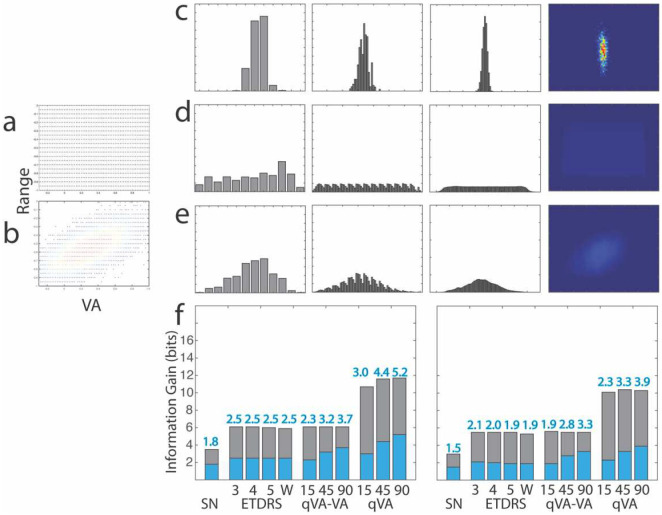
Distributions and information gain in visual acuity tests. (a,b) Distributions of VA threshold and VA range of the simulated observers in Simulations 1 and 2. (c) From left to right: Distributions of the test scores *P*(*y*_*j*_|*x*_*i*_) of one representative simulated observer *x*_*i*_ in the Snellen, ETDRS (3-mistake rule), and qVA test (45 optotypes), with results from the qVA test scored as VA-only, and as both VA threshold and VA range. (d, e) Distributions of the test scores *P*(*y*_*j*_) of the populations in Simulations 1 and 2, respectively. (f) Expected information gain *IG*(*Y*|*X*) of the various tests in Simulation 1 (left) and Simulation 2 (right). For ETDRS, results from the 3-, 4-, 5- mistake and whole chart rules are shown. For qVA, results from testing with 15, 45, and 90 optotypes are shown.

**Table 1.** Entropy, information gain and number of classes from visual acuity tests.

			ETDRS				qVA					
		SNELLEN	# Mistakes			Whole chart	VA only			VA+Range		
			3	4	5		15	45	90	15	45	90
**SIMULATION 1**	H(Y) (bits)	3.5	6.1	6.1	6.0	5.9	6.1	6.1	6.1	10.7	11.6	11.7
	H(Y|X) (bits)	1.6	3.5	3.5	3.6	3.4	3.8	2.9	2.4	7.8	7.2	6.5
	IG (bits)	1.8	2.5	2.5	2.5	2.5	2.3	3.2	3.7	3.0	4.4	5.2
	N(Y|X)	3.6	5.7	5.7	5.6	5.6	5.0	9.1	12.7	7.9	20.5	37.2
**SIMULATION 2**	H(Y) (bits)	3.0	5.5	5.5	5.5	5.3	5.6	5.5	5.5	10.1	10.4	10.3
	H(Y|X) (bits)	1.6	3.5	3.5	3.6	3.4	3.7	2.8	2.3	7.7	7.2	6.5
	IG (bits)	1.5	2.1	2.0	1.9	1.9	1.9	2.8	3.3	2.3	3.3	3.9
	N(Y|X)	2.8	4.2	4.0	3.8	3.8	3.8	6.8	9.5	5.1	9.6	14.6

**Table 2.** Entropy, information gain and number of categories from contrast sensitivity tests.

		PELLI-ROBSON	CVS-1000	qCSF					
				AULCSF			CSF		
				15	25	50	15	25	50
**SIMULATION 1**	H(Y) (bits)	4.0	9.9	5.4	5.4	5.4	16.2	16.0	15.7
	H(Y|X) (bits)	1.7	6.4	2.2	1.8	1.4	8.5	7.8	6.7
	IG (bits)	2.3	3.5	3.2	3.6	4.1	7.6	8.1	9.0
	Categories	5.0	11.1	9.1	12.1	16.7	196.1	281.2	498.0
**SIMULATION 2**	H(Y) (bits)	2.8	7.8	4.9	4.8	4.8	13.9	13.5	12.8
	H(Y|X) (bits)	1.7	6.5	2.7	2.3	1.7	9.4	8.8	7.8
	IG (bits)	1.1	1.4	2.1	2.6	3.1	4.6	4.7	5.1
	Categories	2.1	2.6	4.3	5.9	8.6	23.7	26.0	34.1

## References

[1] Newton I. Philosophiae naturalis principia mathematica. Vol. 1: G. Brookman, 1833.

[2] Ellis B. Basic concepts of measurement. 1968.

[3] Gauch HG Jr, Gauch HG. Scientific method in practice: Cambridge University Press, 2003.

[4] Gower B. Scientific method: A historical and philosophical introduction: Routledge, 2012.

[5] Penrose R. The road to reality: A complete guide to the laws of the universe: Random house, 2005.

[6] Houle D, Pélabon C, Wagner GP, Hansen TF. Measurement and meaning in biology. The quarterly review of biology 2011;86(1):3–34. 21495498 10.1086/658408

[7] Krantz D, Luce D, Suppes P, Tversky A. Foundations of measurement, Vol. I: Additive and polynomial representations. 1971.

[8] Liptak BG. Instrument Engineers’ Handbook, Volume One: Process Measurement and Analysis: CRC press, 2003.

[9] De Vet HC, Terwee CB, Mokkink LB, Knol DL. Measurement in medicine: a practical guide: Cambridge university press, 2011.

[10] Standardization IOf. Accuarcy (trueness and precision) of measurement methods and results—part 1: General principles and definitions. 1994.

[11] International Organization for Standardization. Precision of test methods — Determination of repeatability and reproducibility for a standard test. 1986.

[12] Bland JM, Altman D. Statistical methods for assessing agreement between two methods of clinical measurement. The lancet 1986;327(8476):307–10. 2868172

[13] Kniestedt C, Stamper RL. Visual acuity and its measurement. Ophthalmology Clinics of North America 2003;16(2):155–70, v. 12809155 10.1016/s0896-1549(03)00013-0

[14] Levenson JH, Kozarsky A. Visual acuity change. Clinical Methods: The History, Physical, and Laboratory Examinations 3rd Butterworths 1990. 21250045

[15] Clare G, Pitts JA, Edgington K, Allan BD. From beach lifeguard to astronaut: occupational vision standards and the implications of refractive surgery. British journal of ophthalmology 2010;94(4):400–5. 19465580 10.1136/bjo.2008.156323

[16] Group ETDRSR. Early Treatment Diabetic Retinopathy Study design and baseline patient characteristics: ETDRS report number 7. Ophthalmology 1991;98(5):741–56. 2062510 10.1016/s0161-6420(13)38009-9

[17] Lesmes LA, Dorr M. Active learning for visual acuity testing. Proceedings of the 2nd International Conference on Applications of Intelligent Systems 2019.

[18] Zhao Y, Lesmes LA, Dorr M, Psychophysical validation of a novel active learning approach for measuring the visual acuity behavioral function. Translational vision science & technology 2021;10(1):1-. 10.1167/tvst.10.1.1 PMC7794273 33505768

[19] Ginsburg AP. Contrast sensitivity and functional vision. International ophthalmology clinics 2003;43(2):5–15. 12711899 10.1097/00004397-200343020-00004

[20] Owsley C. Contrast sensitivity. Ophthalmology Clinics of North America 2003;16(2):171–7. 12809156 10.1016/s0896-1549(03)00003-8

[21] Ginsburg AP. A new contrast sensitivity vision test chart. American journal of optometry and physiological optics 1984;61(6):403–7. 6742102 10.1097/00006324-198406000-00011

[22] Pelli D, Robson J. The design of a new letter chart for measuring contrast sensitivity. Clinical Vision Sciences: Citeseer, 1988.

[23] Lesmes LA, Lu Z-L, Baek J, Albright TD. Bayesian adaptive estimation of the contrast sensitivity function: The quick CSF method. Journal of vision 2010;10(3):17-. 10.1167/10.3.17 PMC4439013 20377294

[24] Arditi A. Improving the design of the letter contrast sensitivity test. Investigative ophthalmology & visual science 2005;46(6):2225–9. 15914645 10.1167/iovs.04-1198

[25] Hou F, Lesmes L, Bex P, Using 10AFC to further improve the efficiency of the quick CSF method. Journal of vision 2015;15(9):2-. 10.1167/15.9.2 PMC4581618 26161631

[26] Cover TM, Thomas JA. Information theory and the stock market. Elements of Information Theory Wiley Inc, New York 1991:543–56.

[27] Nyquist H. Certain topics in telegraph transmission theory. Transactions of the American Institute of Electrical Engineers 1928;47(2):617–44.

[28] Hartley RV. Transmission of information 1. Bell System technical journal 1928;7(3):535–63.

[29] Shannon CE. A mathematical theory of communication. The Bell system technical journal 1948;27(3):379–423.

[30] Douglas H. How to measure anything: finding the value of intangibles in business. John Wiley & Sons, 2007.

[31] Larose DT, Larose CD. Discovering knowledge in data: an introduction to data mining. Vol. 4: John Wiley & Sons, 2014.

[32] Lu Z-L, Dosher B. Visual psychophysics: From laboratory to theory: MIT Press, 2013.

[33] Kim W, Pitt MA, Lu Z-L, A hierarchical adaptive approach to optimal experimental design. Neural computation 2014;26(11):2465–92. 25149697 10.1162/NECO_a_00654 PMC4275799

[34] Snellen H. Optotypi ad visum determinandum (letterproeven tot bepaling der gezichtsscherpte; probebuchstaben zur bestimmung der sehschaerfe). Utrecht, The Netherlands: Weyers 1862.

[35] Bailey IL, Lovie-Kitchin JE. Visual acuity testing. From the laboratory to the clinic. Vision research 2013;90:2–9. 23685164 10.1016/j.visres.2013.05.004

[36] Shamir RR, Friedman Y, Joskowicz L, Comparison of Snellen and Early Treatment Diabetic Retinopathy Study charts using a computer simulation. Int J Ophthalmol 2016;9:119–23. 26949621 10.18240/ijo.2016.01.20 PMC4768517

[37] Chen Z, Zhuang Y, Xu Z, Sensitivity and stability of functional vision tests in detecting subtle changes under multiple simulated conditions. Translational Vision Science & Technology 2021;10(7):7-. 10.1167/tvst.10.7.7 PMC8196408 34100925

[38] Thurman SM, Davey PG, McCray KL, Predicting individual contrast sensitivity functions from acuity and letter contrast sensitivity measurements. Journal of vision 2016;16(15):15-. 10.1167/16.15.15 PMC5221673 28006065

[39] Hou F, Lesmes LA, Kim W, Evaluating the performance of the quick CSF method in detecting contrast sensitivity function changes. Journal of vision 2016;16(6):18-. 10.1167/16.6.18 PMC4898274 27120074

[40] Lovie-Kitchin JE. Validity and reliability of visual acuity measurements. Ophthalmic and physiological optics 1988;8(4):363–70. 3253626 10.1111/j.1475-1313.1988.tb01170.x

[41] Dougherty BE, Flom RE, Bullimore MA. An evaluation of the Mars letter contrast sensitivity test. Optometry and Vision Science 2005;82(11):970–5. 16317373 10.1097/01.opx.0000187844.27025.ea

[42] Elliott DB, Sanderson K, Conkey A. The reliability of the Pelli-Robson contrast sensitivity chart. Ophthalmic and Physiological Optics 1990;10(1):21–4. 2330208

[43] Patel PJ, Chen FK, Rubin GS, Tufail A. Intersession repeatability of contrast sensitivity scores in age-related macular degeneration. Investigative ophthalmology & visual science 2009;50(6):2621–5. 19218618 10.1167/iovs.08-2407

[44] Rubin G. Reliability and sensitivity of clinical contrast sensitivity tests. Clin Vision Sci 1988;2(3):169–77.

[45] Thayaparan K, Crossland MD, Rubin GS. Clinical assessment of two new contrast sensitivity charts. British Journal of Ophthalmology 2007;91(6):749–52. 17166891 10.1136/bjo.2006.109280 PMC1955579

[46] Arditi A, Cagenello R. On the statistical reliability of letter-chart visual acuity measurements. Investigative ophthalmology & visual science 1993;34(1):120–9. 8425819

[47] Kheterpal S, Jones HS, Auld R, Moseley MJ. Reliability of visual acuity in children with reduced vision. Ophthalmic and Physiological Optics 1996;16(5):447–9. 8944189

[48] Laidlaw DAH, Tailor V, Shah N, Validation of a computerised logMAR visual acuity measurement system (COMPlog): comparison with ETDRS and the electronic ETDRS testing algorithm in adults and amblyopic children. British Journal of Ophthalmology 2008;92(2):241–4. 17993577 10.1136/bjo.2007.121715

[49] Zhao Y, Lesmes LA, Dorr M, Lu Z-L. Quantifying uncertainty of the estimated visual acuity behavioral function with hierarchical Bayesian modeling. Translational Vision Science & Technology 2021;10(12):18-. 10.1167/tvst.10.12.18 PMC8525832 34647962

[50] Kaiser PK. Prospective evaluation of visual acuity assessment: a comparison of snellen versus ETDRS charts in clinical practice (An AOS Thesis). Transactions of the American Ophthalmological Society 2009;107:311. 20126505 PMC2814576

[51] Zhao Y, Lesmes LA, Hou F, Lu Z-L. Hierarchical Bayesian modeling of contrast sensitivity functions in a within-subject design. Journal of Vision 2021;21(12):9-. 10.1167/jov.21.12.9 PMC8606820 34792537

[52] Rohaly AM, Owsley C. Modeling the contrast-sensitivity functions of older adults. JOSA A 1993;10(7):1591–9. 10.1364/josaa.10.001591 8350148

[53] Watson AB, Ahumada AJ. A standard model for foveal detection of spatial contrast. Journal of vision 2005;5(9):6-. 10.1167/5.9.6 16356081

[54] Zheng H, Wang C, Cui R, Measuring the contrast sensitivity function using the qCSF method with 10 digits. Translational Vision Science & Technology 2018;7(6):9-. 10.1167/tvst.7.6.9 PMC6238983 30479880

[55] Haymes SA, Roberts KF, Cruess AF, The letter contrast sensitivity test: clinical evaluation of a new design. Investigative ophthalmology & visual science 2006;47(6):2739–45. 16723494 10.1167/iovs.05-1419

